# Chitosan and polyvinyl alcohol composite films containing nitrofurazone: preparation and evaluation

**Published:** 2014-01

**Authors:** Maryam Kouchak, Abdolghani Ameri, Basireh Naseri, Sara Kargar Boldaji

**Affiliations:** 1 Nanotechnology Research Center, Ahvaz Jundishapur University of Medical Sciences, Ahvaz, Iran; 2 Department of Pharmaceutics, School of Pharmacy, Ahvaz Jundishapur University of Medical Sciences, Ahvaz, Iran; 3 Microbiology Research Center, Ahvaz Jundishapur University of Medical Sciences, Ahvaz, Iran; 4 Department of Food and Drug Control, School of Pharmacy, Ahvaz Jundishapur University of Medical Sciences, Ahvaz, Iran

**Keywords:** Antibacterial, Chitosan, Nitrofurazone, Polyvinyl alcohol, Wound Dressing

## Abstract

***Objective(s):*** The aim of this study was to insert nitrofurazone in a chitosan membrane to be used as a wound dressing.

***Materials and Methods:*** Several blend films using chitosan (Cs) and polyvinyl alcohol (PVA), containing nitrofurazone were prepared by means of casting/solvent evaporating technique. Different characteristics such as mechanical properties, water vapor transmission rate (WVTR), oxygen permeability (OP), swelling ability (SW), differential scanning calorimetric (DSC), drug release profiles and antibacterial activity of the films were investigated.

***Results:*** The results showed that nitrofurazone decreased tensile strength, OP and SW of Cs films, while increased WVTR. Addition of PVA at any concentration improved mechanical properties, reduced WVTR, and increased OP and SW of nitrofurazone-loaded Cs films. The latter films showed higher activity against *Pseudomonas aeruginosa* than drug-free chitosan films.

***Conclusion:*** The presence of PVA improves many properties of Cs-nitrofurazone films and makes them more desirable as dressing material for burn wounds. Although nitrofurazone alone is ineffective against *P. aeruginosa*, it is able to increase antibacterial effect of chitosan in composite films.

## Introduction

Various formulations of topical products including ointments, creams and wound dressings have been used to treat burn wounds. However, repeated applications of them and frequent wound washing may cause dehiscence of it and results in pain for the patient. Recently, chitosan (Cs) and chitosan products have found wide applications as dressing to protect wound and enhance healing (1). 

Chitosan is a poly-β-(1–4)-D-glucosamine obta-ined by partial deacetylation of chitin and used in its cationic form (2). Presently, chitosan is employed for preparation of drug-loaded films for wound dressing due to its characteristics for film forming properties, homeostasis, biodegradability, biocompatibility, antimicrobial and wound healing activity and its ability to absorb exudates (3, 4).

Several stages take place in burn wounds including infection, necrosis and agglutination followed by proliferation and epidermis formation. Bacterial infections of burn wounds usually delay and complicate the healing process (1).

Various antimicrobial agents have been investing-ated for care and therapy of minor and superficial burns, as well as initial treatment of deeper burns before excision and skin grafting. Nitrofurazone is a potential antibiotic that is administered topically to treat wounds, burns, ulcers and skin infection to combat a wide array of microorganisms and to prepare surfaces before skin grafting (5). However, due to its high permeability through the skin, nitrofurazone remains for a limited time in the location applied.

In addition, antibacterial activity and wound healing properties of chitosan make it a suitable candidate as a dressing to be used in burn and wound care. However, the low mechanical strength of chitosan necessitates the need for water-soluble, non-toxic polymers such as cellulose derivatives poly ethylene oxide (PEO) and poly vinyl alcohol (PVA) to be blended with. In this study, PVA was chosen because of its good mechanical properties, excellent chemical resistance, biodegradability, easy prepara-tion and film forming ability (7-9). The goal of the study was to insert nitrofurazone in a chitosan-PVA composite film in order to decrease nitrofurazone release rate and improve antibacterial effect of the film as a wound dressing.

## Materials and Methods

***Materials***

Chitosan (Cs) with deacetylation degree of 97% and viscosity grade of < 25 cp was purchased from Primex (Iceland). Poly vinyl alcohol (PVA) (MW 72000) was obtained from Merck Co. (Germany). Nitrofurazone was kindly supplied by Behvazan Co. (Iran). All other materials used in this experiment were of analytical reagent (AR) grade.

***Preparation of the films***

The films were prepared using casting and solvent evaporation. Cs was dissolved in acetic acid 1.8% v/v under gentle agitation to produce Cs solution 3% w/v, followed by addition of propylene glycol 1.43% as a plasticizer. In order to prepare drug-loaded Cs films, aqueous solutions of PVA (0%, 2%, 3% and 4%) and nitrofurazone solutions (3 mg/ml) in NaOH 0.3 M were added to equal volumes of chitosan hydrogels followed by stirring for 15 min at room temperature. The resulting mixtures were allowed to stand until air bubbles disappeared, and then 35 ml portions of solution were cast into glass Petri dishes and dried at 40^°^C, overnight. 

After cooling, all films were carefully detached from the glass Petri dishes and stored in airtight desiccators containing saturated magnesium nitrate solution (relative humidity of 50%) until used.

***Evaluation of the films***

*Mechanical properties*

The thickness of the films was measured using a micrometer at five locations and the mean thickness was calculated.

The strain-stress mechanical properties of films were evaluated using a texture analyzer (BERDER Co, China). The test films (2×5 cm^2^ test sections) were held between two clamps at a distance of 3 cm. During measurement, the film was pulled by top clamp at the rate of 10 mm/min. The tensile strength and elongation at break were calculated as follows (10):

Tensile strength (N/mm^2^) = Breaking force (N)/Cross-sectional area of sample (mm^2^)

Elongation at break (%) = [Increasing in length at breaking point (mm)/original length (mm)] × 100

*Swelling degree (Sw)*

The Swelling degree of the films was measured by gravimetric method. The completely dried films (2 × 2 cm^2^) were weighed. Then, they were subme-rged in phosphate buffer solution (PBS) and incubated at 37°C for 24 hr. The resultant swollen films were removed; the excess water was omitted carefully with filter paper and weighed immediately. The swelling degree of the film is the increase in weight, expressed as percentage (11). 

*Water vapor transmission rate (WVTR)*

The films were cut and placed on top of tubes containing 5 g of calcium chloride and held in oven at 50°C in order to achieve constant weights. Then tubes were placed in a desiccator containing a saturated solution of NaCl (75% relative humidity). The vapor penetration was determined by weighing the tubes on day 0, 1, 2, 3, 4 and 5, respectively. Line-ar regression was used to estimate the slope of this line in g/day and WVTR (g/m^2^.day) was calculated by dividing the slope by the area (m^2^) (12). 

*Oxygen permeability (OP)*

Oxygen penetration through films was perform-ed by placing each film on top of open 250 ml-flasks (test area: 1.075 × 10^-3^ m^2^) containing deionized water. The negative and positive controls were the closed flask with an airtight cap and the open flask, respectively. The flasks were placed in an open environment under constant agitation for 24 hr. Dissolved oxygen in water samples were analyzed according to Winkler’s method. OP (g/m^2^.day) was expressed as the amount of oxygen penetration through the film during 24 hr (12). 

*Differential scanning calorimetric (DSC)*

The thermal properties of nitrofurazone, PVA and the chitosan ﬁlms were characterized by a differential scanning calorimeter (DSC, Mettler Toledo CH-8603, Switzerland). Dried samples were exposed to nitrogen gas while being heated between 25 to 300^°^C at the rate of 30^°^C/min.

*In vitro drug release*

Release of nitrofurazone from chitosan films (1.5×1.5 cm^2^) was evaluated by the modified USP dissolution apparatus 2 in 30 ml PBS at 32 ±0.5^°^C. 

**Table 1:** Mechanical properties of chitosan films (mean±SD, n=3)

Formulations	PVA (%)	Thickness(µm)	TS^e^ (MPa)	Elongation (%)
Cs	0	94 ± 11.402	28.369 ± 0.813	20.873 ± 0.69
Cs N^a^	0	106 ± 8.944	4.078 ± 0.813	7.348 ± 0.595
Cs P2 N^b^	2	140 ± 15.811	5.111 ± 0.546	25.984 ± 0.048
Cs P3 N^c^	3	198 ± 13.038	6.481 ± 0.386	48.726 ± 0.264
Cs P4 N^d^	4	254 ± 15.166	6.168 ± 0.301	58.991 ± 0.518

a nitrofurazone-loaded chitosan film

b, c, d nitrofurazone- loaded chitosan/PVA blend film with 3:2, 3:3 and 3:4 Cs:PVA ratio, respectively

e tensile strength

**Table 2:** Swelling degree of films at 24 hr (Mean±SD, n=3)

Formulation		Sw_24_ (%)
Cs		102.58±1.5
Cs N		73.24±1.29
Cs P_2_ N		86.92±8.13
Cs P_3_ N		75.84±3.97
Cs P_4_ N		76.18±8.03

**Figure 1:**
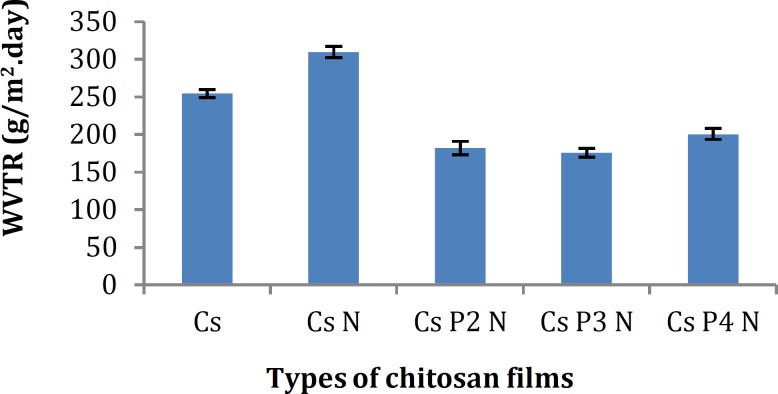
Water vapor transmission rate of different types of chitosan films (mean±SD, n=3)

The rotary paddles were adjusted to 50 rpm. At appropriate time intervals the amount of nitrofurazone released from the drug-loaded films was evaluated by UV spectrophotometer at 377 nm.

*Antibacterial activity*

The zone inhibition test was carried out with a modified agar diffusion assay. The films were cut into 7 mm diameter discs. The discs were placed on Meuller Hinton agar in Petri dishes which had been seeded with bacterial cell suspensions (*Pseudomonas aeroginosa* or *Staphylococcus aureus*) adjusted to Mcfarland’s standard. The Petri dishes were examined for zone of inhibition after 48 hr incubation at 37^°^C. To obtain nitrofurazone paper disc containing equal concentration of drug to the blend films, 10 ml nitrofurazone (3 mg/ml) was added to each paper disc (7 mm-diameter) allowing them to dry at room temperature.

***Statistical analysis***

All experiments were carried out in triplicate and expressed as mean ± SD. Statistical analysis of data was performed using one-way ANOVA.

## Results

***Mechanical properties***

The thickness, tensile strength (TS) and the elongation at break of Cs films are summarized in Table 1. 

As shown in the table, addition of nitrofurazone weakened the mechanical properties of CsN films, significantly (*P*<0.05). On the other hand, as compared to CsN films, the presence of PVA in Cs-nitrofurazone films significantly increased their tensile strength and elongation (*P*<0.05).

**Table 3:** Antibacterial activity of the prepared films and nitrofurazone disc (mean±SD, n=5)

Formulation	Inhibition zone (mm)	
	*Pesudomonas* * aeruginosa*	*Staphylococcus aureus*
N	NE^a^	10.20 ± 1.30
Cs	22.60 ± 6.23	NE
Cs N	25.80 ± 4.02	8.20 ± 0.84
Cs P2 N	Whole Petri dishes	NE
Cs P3 N	21.20 ± 1.79	NE
Cs P4 N	8.00± 0.5	8.00 ± 1.00

a NE: inhibition zone not exhibited

**Figure 2:**
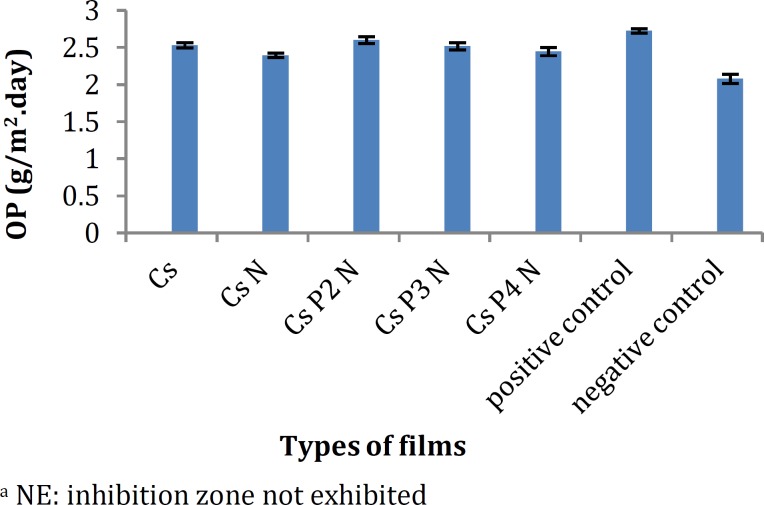
Oxygen permeability of the chitosan films (mean±SD, n=3)

***Swelling degree (Sw)***

Table 2 shows the water uptake of the films after 24 hr. Cs films showed the highest increase in swelling degree, while Nitrofurazone caused a significant decrease in this value (*P*<0.05).

***Water vapor transmission rate (WVTR)***

As seen in Figure 1, the water vapor transmission rate across the Cs films is relatively high. Cs- nitrofourazone films showed higher WVTR. 

***Oxygen permeability (OP)***

The oxygen permeability of the films is presented in Figure 2. The dissolved oxygen value in all flasks covered with the composite films was significantly higher than that of the negative control (2.08 ± 0.062 g/m^2^.day) (*P*<0.05) and a slightly lower than that of the positive control (2.726 ± 0.029 g/m^2^.day).

***Differential scanning calorimeter (DSC)***

**Figure 3:**
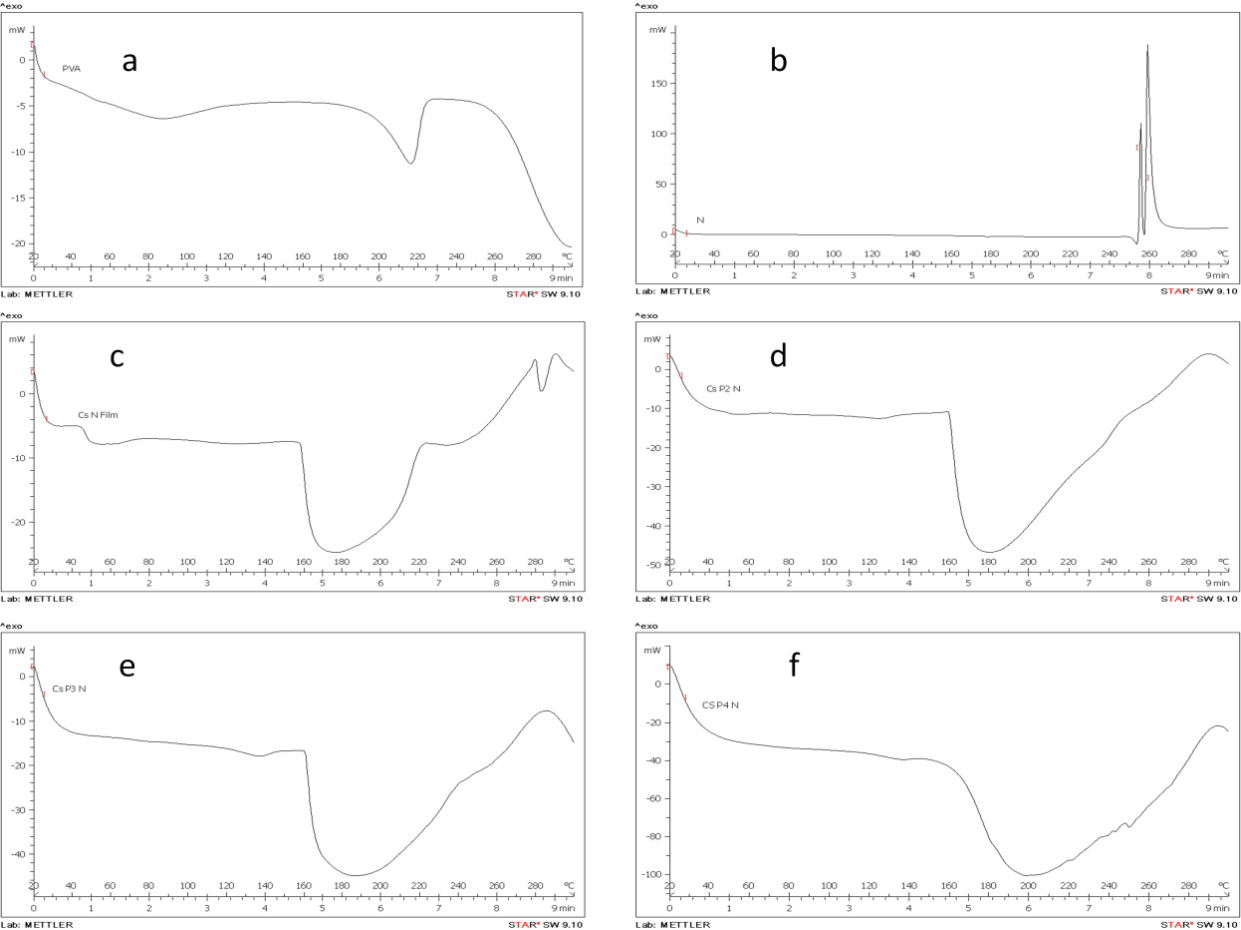
DSC thermograms: a: PVA, b: Nitrofurazone, c: CsN film, d: CsP2N film, e: CsP3N film, f: CsP4N film

Thermogram of pure nitrofurazone showed two exothermic peaks at 255 and 259^o^C. PVA powder and Cs-nitrofurazone films showed endothermic peaks at 216 and 176^o^C, respectively. In the thermogram of blend films (Figure 3), only one broad endothermic peak was observed between the above two temperatures that moved to higher levels with increasing PVA proportion in films. 

***In-vitro drug release***

**Figure 4:**
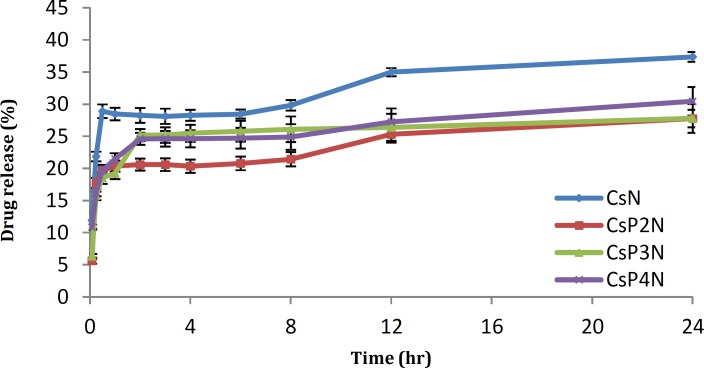
Profile of nitrofurazone release from different Cs-PVA blend films in PBS (Mean ±SD, n=3)

As can be seen in Figure 4, a burst release of drug occurred during the first 30 min but then the rate of drug release slowed down and continued overnight. Cs -PVA films showed lower burst release. 

***Antibactial activity***

As displayed in Table 3, nitrofurazone effectively inhibited the growth of *S. aureus*, but did not inhibit the growth of *P. aeroginosa*. Conversely, chitosan markedly affected *P. aeroginosa* but was ineffective against *S. aureus*. However, CsN films showed antibacterial effect against both microorganisms.

## Discussion

Elasticity and strength of dressing materials (films) are among the primary factors that protect the wound surfaces from external factors (13). In this study, the mechanical properties of Cs films were significantly deteriorated by addition of nitrofurazone to the formulations. These poor mechanical properties may be related to the structural disarrangement and discontinuity of Cs film caused by incorporation of the drug. These results are in line with those reported by other authors when adding other nonpolymeric material to a chitosan matrix (14-16).

In our study, the addition of PVA to Cs films containing nitrofurazone significantly increased their elongation and tensile strength (*P*<0.05). Previous reports showed that the blends of polysaccharides with different ratios of polymers enhance the tensile properties of dressing films (17-18). The high tensile strength of Cs films may be explained in terms of formation of intermolecular hydrogen bonding between the –NH_3_^+^ of the Cs backbone and –OH^-^ of PVA. Destruction of the ordered structures of PVA molecules in the acetic acid solution, results in the -OH^−^ groups being exposed to readily formed hydrogen bonds with protonated amino groups (-NH_3_^+^) of the Cs (18).

Although the tensile strength of the films was improved by addition of PVA, but it was not concentration dependent (*P*>0.05).

For wound healing, water preserving ability of the films is very important in skin tissue engineering (2). Possession of polymer bearing amine (-NH_2_) and hydroxyl groups (-OH^-^) increases chitosan affinity to water and hydrogen bond formation with hydrophilic solvents (19). Blending of Cs with less hydrophilic materials (nitrofurazone) decreases the hydrophilicity of Cs and causes a reduction in water absorption (15, 20). Addition of PVA as a hydrophilic polymer to chitosan-nitrofurazone films resulted in significantly higher water uptake (*P*<0.05). Although it was reported that the moisture content increased with increase in PVA concentration in chitosan-PVA blend films (7), this study did not show such correlation(*P*<0.05). 

An ideal dressing is the one that controls the evaporative water loss from a wound at an optimal rate. Permeability of moisture through the film for wound dressing is important to keep the wound comfortable and promote the healing process (21). It was reported that the film must be permeable to the extent that a moist exudates under dressing is maintained; to inhibit excess fluid absorption and evaporation leading to desiccation of the wound bed (10). 

The evidence from this study suggests that the addition of nitrofurazone to Cs films causes a reduction in strength of intermolecular bonds in primary structure of chitosan which could be responsible for the decrease of barrier properties of the film against water permeation. 

The strong intermolecular interactions between chitosan and PVA molecules result in shorter intermolecular distances forming more compact films. Moreover, hydrogen bond interactions between the two polymers reduce the availability of the hydrophilic groups, leading to a decrease in their interactions with water molecules, therefore, reducing the water vapor transmission rate (22-24).

Generally, hydrophilic biopolymer films are good oxygen barriers. From the results, it can be concluded that oxygen was able to penetrate through all the composite films. Presence of propylene glycol in these films helps the movement of polymer chains to allow passage of oxygen molecules. The relative affinity of the hydrophilic propylene glycol as a plasticizer has been reported to be signiﬁcant (25,26). In their study, Wittaya-areekul and Prahsarn found that increasing the concentration of propylene glycol from 0.5 to 1.5% (w/v) in the chitosan–polysaccharides composite ﬁlms had a tendency to increase oxygen penetration through the films (11). Our results showed an inverse relationship between water-vapor transmission and oxygen permeability which is in agreement with the findings of others (27). 

DSC thermogram of pure nitrofurazone showed two exothermic peaks at 255 and 259^°^C which may be attributed to the degradation of the drug. In this study, CsN films showed a very broad endothermic peak at 176^°^C which might be related to the glass transition temperature (Tg) of Cs film. Our findings are in line with the study of Yinyong Li et al. who reported a small transition area at the range of 160 -170^°^C on DSC thermogram of a glycerol-plasticized Cs film (28). In other studies, higher value of Tg (205^°^C) reported for unplasticized Cs films. This difference could be attributed to the effect of plasticization by glycerol or propylene glycol that was used in our study (29, 30).

We found an endothermic peak at 216^°^C for PVA powder which agrees well with the finding of Kenawy *et al *who reported T_m_ of 217^°^C for virgin PVA (31).

In each chitosan-PVA composite film containing nitrofurazone, only one wide endothermic peak was observed between 176 and 216^°^C that shifted to higher levels with increasing PVA proportion in films. The changes of Tm with respect to that of pure PVA suggest the miscibility and interphase interaction between the components of a polymer blend (19). This miscibility can be attributed to hydrogen bonding between hydroxyl groups of PVA and the partially protonated amine groups of chitosan. 

Drug release test showed a burst release of drug during the first 30 min but then the rate of drug release slowed down and continued overnight. However, Cs -PVA films showed lower burst release. The strong intermolecular interactions between chitosan and PVA molecules resulted in more cross-linked regions in films that showed as impenetrable barriers to the movement of drug molecules. It seems that the drug release continues by polymer gradual erosion. When the ratio between Cs and PVA was 3:2 (in CsP2N formulation), the blend ﬁlms showed the lowest drug release rate. This result may be attributed to the increase in PVA which causes a decrease in compatibility and enhancing electrostatic repulsion between Cs and PVA. Similar results were reported by Rao et al. who found that the highest cross linking takes place between Cs and guar agar at the lowest volume ratio used in their study (18). 

Nitrofurazone is a nitrofuran derivative with a broad spectrum of antibacterial activity with weak activity against *Pseudomonas *spp. (9)*.* As expected, in this study, nitrofurazone effectively inhibited the growth of *S. aureus*, but did not inhibit the growth of *P. aeroginosa*. Conversely, chitosan was markedly effective only against *P. aeroginosa*. However, CsN film showed antibacterial effect against both microorganisms.

The exact mode of action of the antimicrobial potential of chitosan is not well understood. It is believed that –NH_3_^+^ groups of Cs binds to the anionic groups of the bacterial cells leading to disruption the outer layer of the cell wall. As for bacteria cell structure, Gram-negative cell wall is made of a thin layer of peptidoglycan and an outer layer consisting of lipopolysaccharide, lipoprotein and phospholipids. It is thought that the protonated amino groups of chitosan at acidic conditions can react with the anionic carboxyl and phosphate groups of the bacterial surface and accordingly inhibit the growth of Gram-negative bacteria. On the other hand, the cell wall of *S. aureus* is mainly composed of peptidoglycan, which does not allow the formation of a surface layer (32, 33). 

The agar diffusion method relies on the diffusion of the compound tested through water-containing agar medium. The diffusion of the drug is dependent upon the size, shape and polarity of the antibacterial material; chemical structure and the cross-linking level of the ﬁlm (34). In this study, the addition of PVA to the formulations reduced the antibacterial effect of the films. This may be attributed to high cross-linking level of Cs-PVA blend films that prevents the diffusion of the drug through the agar medium. However, weak inhibition of *S. aureus* growth was observed by CsP4N film containing higher concentration of PVA that is probably due to higher rate of drug diffusion. This is in agreement with the data mentioned in the drug release section indicating formulations with higher concentration of PVA exhibited higher rate of drug release in the dissolution medium than those with lower concentration.

## Conclusion

Composite ﬁlms of chitosan containing nitrofurazone were developed for wound dressing applications. Although nitrofurazone is ineffective against *P. aeruginosa*, the chitosan films containing nitrofurazone showed a significant inhibitory effect against the growth of this microorganism which was even higher than that of the drug-free chitosan films. This is the benefit of application of Cs as a carrier for nitrofurazone in treatment of burn wounds. As *P. aeruginosa* remains a cause of serious wound infection and mortality in burned patients, clinical trial is proposed to evaluate the usability of the films.

*In vitro* evaluation revealed that PVA can be incorporated into chitosan ﬁlm to improve its mechanical properties while substantially maintaining good vapor penetration, water swelling, and oxygen penetration properties. These properties are desirable for burn wound dressing materials.
